# Editorial: Control of Presynaptic Function by Axonal Dynamics

**DOI:** 10.3389/fncel.2019.00543

**Published:** 2019-12-06

**Authors:** Federico F. Trigo, Shin-ya Kawaguchi

**Affiliations:** ^1^CNRS UMR8003, SPPIN Laboratory, Cerebellar Neurophysiology Group, Faculté des Sciences Fondamentales et Biomédicales, Université de Paris, Paris, France; ^2^Departamento de Neurofisiología Celular y Molecular, Instituto de Investigaciones Biológicas Clemente Estable, Montevideo, Uruguay; ^3^Department of Biophysics, Graduate School of Science, Kyoto University, Kyoto, Japan

**Keywords:** axonal dynamics, synaptic release, action potential (AP), imaging, patch-clamp

Neurons are highly polarized cells: they possess a dendritic arbor, which is classically considered the input compartment of the cell; a cellular body, where incoming information is integrated; the axon, which is a transmission compartment and classically considered as a conducting line responsible for action potential (AP) propagation; and axonal varicosities, which are the output compartments and where transmitter release occurs. Axons of mammalian brain neurons are highly diverse: some are myelinated and others unmyelinated, some are only a few hundred microns long and others more than 1 m long, some are highly branched and others almost unbranched, etc. The same is true for the presynaptic terminals: some are extremely big, packing hundreds of individual synaptic release sites, while others possess only a single active zone. This morphological diversity is accompanied by molecular or subcellular diversity. The distribution and density of ion channels vary among different axonal locations: some are highly concentrated in the AIS (axon initial segment; Kole et al., 2007), for example, and others in presynaptic terminals (Rowan et al., 2016), which also possess a variety of receptors for different neurotransmitters and/or neuromodulators.

Although it is safe to assume that at some level all axons behave similarly, ignoring axonal diversity would run the risk of oversimplifying axonal function and misunderstanding axonal physiology. However, studying the axonal compartment and presynaptic terminals is often challenging because of the features that were already mentioned: axon and presynaptic terminals are usually small structures (in the sub-micrometer range), they function extremely rapidly, and they have a complex morphology. Understanding the physiology of these tiny structures therefore requires the development of new techniques and the contribution of different methodologies.

Numerous reviews have been published in the last decade highlighting the computational capabilities of the axon (Debanne, 2004; Debanne et al., 2011), notably in terms of its effects on transmitter release. The scope of this “Research Topic” is to bring together different researchers studying the axonal compartment and presynaptic terminals in order to document recent progress and, more importantly, to highlight that the function of the axon is more complicated than previously thought and that much remains to be done in order to understand axonal function.

Two of the reviews treat the rapidly growing topic of axonal analog signaling—soma-axon interactions in the subthreshold voltage range. In the CNS, information transfer is classically considered to be digital, but subthreshold voltage signals can propagate along long distances and they can affect neurotransmitter release by modulating presynaptic voltage-gated channels. In one review, Zbili and Debanne describe analog signaling both from a historical and from a physiological perspective, as well as analog signaling mechanisms and their potential benefits for network computation. In the other review, Trigo discusses another, less-studied form of analog transmission, whereby subthreshold voltage signals backpropagate from the axon toward the soma. This so-called antidromic analog transmission challenges the dynamic polarization theory of Ramón y Cajal, according to which information propagates unidirectionally from dendrites to soma, and from soma to axon.

In another study, Yang et al. perform electrophysiological recordings from the soma and axonal blebs of the AIS in midbrain dopaminergic neurons (substantia nigra pars compacta and ventral tegmental area) and compare the biophysical properties of voltage-gated sodium channels in the two sites. Although the general properties of axonal and somatic channels are similar, axonal channels inactivate at more hyperpolarized potentials and more slowly than somatic channels. Surprisingly, associated immunostaining results show that the AIS of dopaminergic cells only expresses the Nav1.2 sodium channel subunit, and not the other typical AIS subunits, Nav 1.1 and 1.6. These results indicate that in some cells, like the dopaminergic cells studied here by Yang et al., the exclusive expression of the Nav1.2 subunit ensures stable AP generation at the AIS.

The AP waveform at the release site is a key factor for neuronal communication because it influences neurotransmitter release and synaptic plasticity. Apart from the AP amplitude and time course, the after-spike potential (the membrane potential, either depolarizing or hyperpolarizing, after the AP) may have a strong impact on axonal excitability. Recently, Zorrilla de San Martin et al. (2017) showed that the after-spike depolarizations induced by the activation of axonal GABA_A_ auto-receptors in Purkinje cells increase the presynaptic Ca^2+^ current and hence release, and have a dramatic effect on short-term plasticity. In a “Theory and Hypothesis” article, Raastad examines the after-spike potential in CNS neuronal thin axons in terms of its mechanisms and functional implications. By simulating such a depolarizing after-spike potential in hippocampal mossy fibers, Kamiya demonstrates in another article the substantial contribution of the passive spread of the AP itself into neighboring axonal regions to the after-spike depolarization.

In addition to the electrical properties of the axon, the mechanisms that control the intracellular Ca^2+^ by internal buffers tightly control transmitter release from presynaptic terminals. In a theoretical paper, Nakamura studies the effects of the calcium chelator EGTA on transmitter release. EGTA has been widely used by neurophysiologists to estimate the coupling distance between the calcium entry site (calcium channels) and the calcium sensor in presynaptic terminals. Contrary to what is usually expected, in the close proximity of the calcium channel EGTA can affect release if the sensor is not saturated, as may happen for brief calcium influxes like those induced by APs. Nakamura's paper emphasizes the importance of measuring the AP characteristics with direct axonal recordings.

In an elegant study, Bolshakov et al. describe the role of a Ca^2+^ binding protein, calretinin, on synaptic transmission with a combination of molecular biology and electrophysiology methods. The authors infect newborn pups' somatosensory neurons with an AAV vector in order to exogenously induce calretinin expression in cells that do not normally express the protein. They then compare synaptic release in infected and non-infected cells. With this approach Bolshakov et al. show that calretinin changes short-term plasticity in cortical synapses.

Miki discusses how Variance to Mean analysis is used to understand synaptic release mechanisms, particularly at high frequency repetitive stimulations. In his study, Miki explains how fluctuations in the number of released vesicles per AP and in the cumulative number of released vesicles during an AP train can be used to distinguish between different docking (or release) site models. In his perspective, Miki also discusses how this method could be complemented by techniques that do not rely on electrophysiological recordings to assess release but rather on direct visualization of synaptic vesicle exocytosis and of vesicle movements, including super-resolution imaging (see, for example, the study of Funahashi et al. in this issue and also Midorikawa and Sakaba, 2017) and electron microscopy (Kusick et al., 2018) techniques.

Two of the research papers describe techniques for high resolution visualization of the dynamics of synaptic vesicle fusion. In an original research article, Funahashi et al. induce the formation of active zone like structures on a glass coverslip in order to image the fusion of synaptic vesicles with total internal reflection microscopy, a super-resolution technique that uses the evanescent field of an angled laser beam. This methodological tour de force allowed the authors to image single vesicle fusion and to follow vesicular movements, and will certainly be used in the future to study presynaptic physiology. In the second technical paper, Dobson et al. describe in detail a simple photoconversion setup that allows seeing the ultrastructure of presynaptic terminals with the high resolution of an electron microscope.

As already mentioned, axonal studies require the development of new techniques. Panzera and Hoppa review in detail the use of genetically-encoded voltage indicators for the study of axonal physiology. Imaging the axon with voltage indicators allows recording from multiple axonal locations simultaneously, fulfilling a goal that cannot be attained with electrophysiological techniques. Although voltage-sensitive fluorescent proteins lag behind electrophysiological techniques in terms of temporal resolution, their development is rapidly advancing and they will probably outcompete electrophysiological methods not only for axon studies but for neurophysiology in general.

Finally, Kawaguchi reviews the myriad of factors that may influence transmitter release from small CNS presynaptic terminals, notably by describing recent literature based on direct patch-clamp recordings from axonal varicosities. This review nicely summarizes this topic by highlighting the complex interactions existing between the axon and its synaptic terminals and how these interactions influence neuronal output and plasticity.

The aim of this “Research Topic” was to highlight recent advances in the field of axonal function, both from the physiological and methodological perspectives. More importantly, we intended to attract the reader's attention about the fact that much remains to be done in order to understand axonal function. We hope we have fulfilled this goal. We would like to thank all the contributing authors and hope the readers will enjoy diving into the study of axonal function.

## Author Contributions

All authors listed have made a substantial, direct and intellectual contribution to the work, and approved it for publication.

### Conflict of Interest

The authors declare that the research was conducted in the absence of any commercial or financial relationships that could be construed as a potential conflict of interest.
